# Mitigating Human Impacts on Wild Animal Welfare

**DOI:** 10.3390/ani13182906

**Published:** 2023-09-13

**Authors:** David W. Macdonald

**Affiliations:** The Wildlife Conservation Research Unit (WildCRU), Department of Biology, University of Oxford, Recanati-Kaplan Centre, Tubney House, Abingdon Road, Tubney OX13 5QL, UK; david.macdonald@biology.ox.ac.uk

**Keywords:** biodiversity conservation, human impacts, vertebrate wildlife, wild animal welfare, wildlife management

## Abstract

**Simple Summary:**

This paper considers examples of how humans negatively affect the welfare of *wild* animals, how such impacts might be reduced and what additional research is needed, including examples from biodiversity conservation, wildlife management, wildlife tourism and wildlife trade. Further, it discusses the relationship between wild animal welfare and biodiversity conservation, including consideration of various ethical viewpoints.

**Abstract:**

Human activities negatively impact the welfare of wild vertebrates in many different contexts globally, and countless individual animals are affected. Growing concern for wild animal welfare, especially in relation to conservation, is evident. While research on wild animal welfare lags behind that focused on captive animals, minimising human-induced harm to wild animals is a key principle. This study examines examples of negative anthropogenic impacts on wild animal welfare, how these may be mitigated and what further research is required, including examples from wildlife management, biodiversity conservation, wildlife tourism and wildlife trade. Further, it discusses the relationship between animal welfare and biodiversity conservation, and synergies that may be achieved between these. Ultimately, it is discussed how the welfare of wild animals may be balanced with other priorities to ensure that welfare is afforded due consideration in interactions between people and wildlife.

## 1. Background

The aim of this paper is to explore harmful human effects on wild animal welfare, propose mitigation strategies, and highlight the connection between animal welfare and biodiversity conservation across various contexts such as wildlife management, tourism and trade. It emphasises the need to attend to animal welfare alongside other concerns in human–wildlife interactions. In covering such a wide scope, there is, unsurprisingly, extensive literature that could be incorporated. Although I have sought to cite the most pivotal work from whatever source, I considered it timely to reflect on the fifty years’ experience of my own unit (WildCRU, University of Oxford) on this topic, and so I have deliberately chosen to draw heavily on that corpus of work which I believe, hopefully unbiasedly, has contributed insight to the topic.

Populations of wild animals are important, and their biology is an emergent property of the individuals that comprise them. That importance impacts almost every aspect of the human enterprise, from agriculture and food security, epidemiology to tourism, in wildlife management and every transdisciplinary aspect of biodiversity conservation. Additionally, on each of these diverse dimensions, interactions with populations intersect with impacts on individuals. The issues arising from human impacts on wild vertebrate welfare are the topic of this critical review. This review considers how these may be mitigated and what further research might lead to reduced impacts. Included are examples where the species is viewed as problematically abundant or scarce, as well as consideration of wildlife tourism and wildlife trade, including that involving free-ranging and captive members of wild species, but excluding animals in zoos or farms as most are likely to have been captive bred for generations. This wide array of examples supports the discussion of the relationship between animal welfare and biodiversity conservation and how the welfare of wild animals may be balanced with other priorities to ensure welfare is properly considered in interactions between people and wildlife.

Human activities negatively impact the welfare of countless wild vertebrates every year. The facets of the human enterprise that cause those impacts are legion, and measures are diverse and varied. Of these, the metric of numbers impacted is perhaps the most straightforward to assess, and to get a sense of the enormity of these numbers consider just one of several sectors: agriculture, which globally is estimated to lead to the deaths, and associated suffering, of billions of wild vertebrates annually [1] (Fischer and Lamey 2018). Attempts to control rodents in Europe alone kill 10–100 million of them annually: any lack in perceived charisma of the species involved is surely counter-balanced by the flabbergasting numbers of individuals whose welfare is impacted. One might wonder whether deaths by the same means of comparably sized mammals of different taxa, say bush babies (which look cuddly), or of colourful birds (which do not look drab), or of gymnures or sengis (despite their having naked tails), would be accepted with similar nonchalance, although neither size, taxon, drabness nor furry tails is thought to be associated with vertebrates’ capacity to suffer. Conservation, and its close cousin, wildlife management, operate in arenas ranging from human–wildlife conflict, harvest and trade (legal and illegal, from medicinal to culinary to pet-keeping), epizootiology, tourism (from animal attractions to photo- to hunting-tourism). It is hard to think of any of these arenas in which animal welfare is not a dimension meriting consideration within the transdisciplinary holism that is or, according to Macdonald [2] (2019), should be, modern conservation. For example, suffering is surely an aspect of the experience of the over 900,000 wild mammals, birds and reptiles that are ‘extracted’ for bush meat annually in just the Sanaga-Cross region of Cameroon and Nigeria [3] (Fa et al. 2006)—some, such as cane rats, may be unpopular and numerous, while others, such as gorillas, both charismatic and imperilled, are global ambassadors for conservation and surely merit individual respect. Further, hundreds of thousands of wild animals are impacted globally by wildlife tourism [4] (Moorhouse et al. 2015a). These and many other effects are set to escalate further as human populations, their wealth, and deeply trodden footprints, grow [5] (Kirkwood 2013).

Welfare impacts may be lethal or non-lethal, and occur either as: (a) unintended side-effects of deliberate actions, such as managing species to protect human or animal health, agriculture, property or biodiversity conservation interests, managing invasive species, hunting animals for food or sport or marking them for research; or (b) through deliberate or unintended change to the environment, such as through the destruction or degradation of habitat, residential or commercial development, environmental pollution or introduction of infectious disease [5,6] (Fraser and Macrae 2011; Kirkwood 2013).

What is animal welfare? Mellor [7] (2016) proposes that an animal’s welfare reflects how it is experiencing its life, and good welfare occurs when an animal’s nutritional, environmental, health, behavioural and mental needs [8] (Mellor and Reid 1994), and its wants [9] (Dawkins 2012), are met. Of course, under this type of definition, the welfare of many wild animals may naturally be far from good, but that is not the topic of this essay, which focuses on that proportion of negative impacts on wild animal welfare that is caused by people, particularly to vertebrates. It is increasingly recognised that all vertebrates are sentient [10] (Veterinary Record Editorial. 2020), that is, able to experience feelings such as pleasure, pain and fear [11,12] (Beausoleil 2020, House of Lords 2021) and that this extends beyond mammals and birds [13] (e.g., Lambert et al. 2019, for reptiles). Indeed, the United Nations tenth annual report on Harmony with Nature published in 2020 (A/75/266), stated (Para 42), ‘*A first step to recognizing the rights of Nature is the recognition that non-human animals are sentient beings, not mere property, and must be afforded respect and legal recognition.*’

Bruckner [14] (2020) discusses different conceptions of animal welfare and the methodologies used to justify them, arguing that philosophical methodology relying on conceptual analysis is crucial to this debate, as any conception of animal welfare is inherently normative. The paper distinguishes between different types of values or norms and suggests that empirical results about folk conceptions of animal welfare can help with conceptual philosophical investigation into the competing conceptions of animal welfare. Coghlan [15] (2022) responds to Bruckner stating that animal welfare is a type of normative value distinct from ethical value, and that ethical reflection and dialogue can help conceptualise animal welfare, arguing that ethical responses can expose existing hidden or denied beliefs about prudential value/well-being and that some judgments about well-being’s nature are internal to, and revealed in, ethical judgments. Coghlan suggests that understanding prudential value requires sensitivity and responsiveness somewhat similar to that required in good ethical thinking; therefore, ethics (and philosophy) can help conceptualise animal welfare.

How are the welfare impacts of people on wild animals identified? Suggestions include the intensity and duration of suffering [16,17] (Broom 1999, Sharp and Saunders 2011), the capacity for the animal to suffer and the number and proportion of individuals affected [18] (Littin et al. 2004). Welfare impacts can therefore be minimised by reducing the number of animals affected and by opting for actions with the least impact. Problems of animal welfare are likely to be lessened by acting early before the population whose members will be affected has either grown or declined problematically [19] (Cowan and Warburton 2011). Further, Hampton, Fisher, and Warburton [20] (2020) caution that the terms humane and inhumane are prone to disingenuous use, for example, to deflect public scrutiny or imply discredit. To avoid all these pitfalls, this essay refers, without prejudgment, to welfare impacts.

The welfare of wild vertebrates is clearly important for the individual animals impacted, as well as for those humans who value them intrinsically. However, events that adversely impact the welfare of individual wild animals may also ultimately threaten the viability of populations [21] (Beausoleil et al. 2018). Hence, in addition to the powerful logic that individual vertebrates have ‘interests’ (in avoiding suffering and in surviving) and intrinsic value, another and practical reason why conservationists may be vigilant regarding the welfare of individual animals is its potential as an early warning for the security of populations. Mention of value is a reminder that ethical frameworks differ (e.g., [22] Vucetich et al. 2021), but many converge on the conviction that where humans have negative impacts on wild animal welfare there is a moral duty to minimise these impacts wherever possible. However, in order to minimise negative welfare impacts, these first need to be identified and understood. This prerequisite has been neglected to the extent that concern for the welfare of wild vertebrates has lagged behind that for captive animals, despite their similar capacities to suffer [23,24] (Sainsbury et al. 1995, Littin 2010); consider how differently Western cultures treat wild wolves (*Canis lupus*) compared to pet dogs (*Canis lupus familiaris*) [25] (Fraser 2008), and wild rats (*Rattus* spp) compared to their laboratory and companion conspecifics [26,27] (Berdoy and Drickamer 2007, Macdonald et al. 2015a).

Exploring the welfare of wild invertebrate animals presents particular challenges, which include limited public awareness and understanding. The evolving literature, particularly on cephalopods [28,29] (Birch et al. 2021; Browning & Birch 2022), highlights their potential sentience broadening questions about wild animal welfare and challenging traditional views on invertebrate welfare [30,31] (Horvath et al. 2013; Carere & Mather 2019). Balancing conservation efforts with ethical considerations is crucial in ensuring respectful treatment of all sentient beings, expanding understanding of welfare beyond vertebrates.

Why has concern for the welfare of wild animals lagged behind? Perhaps because of the habit of custom or tradition, or because of a lack of awareness and information about anthropogenic impacts on wild animal welfare or because free-ranging animals are not perceived as being under human stewardship in the way that captive animals are [32] (Kirkwood et al. 1994). The suffering of so-called ‘pest’ animals has routinely been particularly under-valued [33] (Mathews 2010b). This may be because ‘pests’ are valued less than other animals, people or the environment, or the utilitarian metric that any harm caused to them can be justified by the damage they are believed to have caused. Pleasingly, addressing wild animal suffering is gaining traction, both in the literature (e.g., [34] Faria & Horta, 2019) and in the formation of such bodies as the Wild Animal Initiative (https://www.wildanimalinitiative.org/, accessed on 1 August 2023), whose aim is to advance ‘understanding of wild animal well-being’.

The philosophy of compassionate conservation emphasises four tenets: do no harm, individuals matter, inclusivity of individual animals, and peaceful coexistence between humans and animals. However, critics argue that the examples presented by compassionate conservationists focus too much on mammals, arbitrarily favour charismatic species such as large predators and megaherbivores, and offer ineffective conservation solutions [35] (Hayward et al., 2019). Critics also argue that compassionate conservation may hinder other forms of conservation, such as translocations and fertility control. Despite these criticisms, a recent review [36] (Coghlan & Cardilini 2022) of the debate surrounding compassionate conservation argues that it is a necessary interdisciplinary dialogue about ethics, values, and conservation. The review identifies 52 specific criticisms from 11 articles and closely examines 33 of them, finding that some criticisms are question-begging, confused, or overlook conceptual complexity. While some issues need to be addressed, such as the differential intrinsic moral value of different sentient animals, the review provides a clearer basis for ongoing interdisciplinary dialogue about the moral value of sentient animals, conservation, and science.

The flushing out of inconsistency, even hypocrisy, advances with growing concern for wild animal welfare [33] (Mathews 2010b) and this is reflected in legislative change. For example, in the late 1990s, New Zealand introduced the Animal Welfare Act (1999), which provides some protection to wild animals, and Europe signed the Agreement on International Humane Trapping Standards with Canada, Russia and the United States. In the 1990s and 2000s, the UK introduced several Acts to protect the welfare of wild animals [37] (Baker et al. 2016) and the UK Animal Welfare (Sentience) Act [12] (House of Lords 2021) is currently being implemented. Most of the relatively sparse wild animal welfare research to date relates to wildlife management [16,17,18,38,39,40,41,42,43] (Broom 1999, Mason and Littin 2003, Littin et al. 2004, Iossa et al. 2007, Sharp and Saunders 2011, Baker et al. 2012, Littin et al. 2014, Hampton et al. 2016b, Baker et al. 2022), but research has also begun to address welfare impacts in wider conservation contexts, such as reintroductions [44] (Harrington et al. 2013), wildlife tourism [4,45,46] (Christiansen et al. 2014, Moorhouse et al. 2015a, Teerlink et al. 2018), wildlife trade [47] (Baker et al. 2013), the exotic pet trade [48,49] (Bush et al. 2014, D’Cruze et al. 2020) and agriculture [33] (Mathews 2010b), as well as impacts on wild animal welfare as covered by the media [50] (Feber et al. 2017).

Martin et al. [51] (2016) explore how the conservation sector can incorporate concerns for social justice, particularly recognition, which involves respecting local knowledge and cultures. The authors identify four components of recognition as an analytical framework, and apply it to explore four traditions of thinking about recognition. Three case studies of conservation conflicts illustrate different theoretical perspectives. The authors conclude that conservation should go beyond distributive justice to incorporate concerns for social recognition and equality of status for local conservation stakeholders, requiring reflection on working practices and intercultural engagement.

A framework to promote procedural justice in conservation decision making has been developed [52] (Ruano-Chamorro et al. 2022) based on 11 criteria, organised into three key domains: Process properties, Agency of participants, and Interpersonal treatment. Seven policy levers can be used to enhance procedural justice. However, addressing broader structural power inequalities and plural and situated conceptions of procedural justice are necessary challenges to overcome. New conservation approaches such as compassionate conservation and multispecies justice challenge conventional norms, aiming to include non-human animal perspectives. A comprehensive conservation ethic should promote an ethics of care and, according to the conclusions of Santiago-Ávila & Lynn 92020 [53]), the codification and enforcement of animal claims.

Although this review highlights the negative impacts humans have on wild animal welfare—hopeful that these can be mitigated—humans do, of course, also provide vital welfare benefits to non-human animals, notably pets and livestock. Consideration of these topics, and of some emerging trade-offs, lead to philosophical topics beyond the scope of this essay, but for completeness I mention that through responsible care, pets receive companionship, medical attention, and safe environments; in the same vein, livestock can receive healthcare, and protection from predators and harsh elements. Amongst the thorny trade-offs, beset with incommensurables, permeating this topic, lethal control of pests, such as rats and rabbits, can improve the welfare of other wild animals, and advance conservation efforts, protecting endangered species and promoting biodiversity. Tilman et al. (2017 [54]) conclude that by managing over-populations, humans prevent habitat destruction and competition for resources, preserving ecosystems for native species, as well as curtailing disease transmission that harms both wildlife and domestic animals.

## 2. Impacts of Wildlife Management on Animal Welfare

Motivations for managing wild vertebrates span attempts to limit negative impacts of wildlife on human and animal health, property, agriculture and economic interests, often involving reducing wildlife numbers, and to limit the negative impacts of humans on wildlife, often involving increasing their numbers [41] (Littin et al. 2014). While we consider the many millions (possibly even billions [1] (Fischer and Lamey 2018)) of rats and mice estimated to be killed globally each year as ‘pests’ [38] (Mason and Littin 2003), bear in mind that a single South African farmer can kill 50 jackals a year [55] (Humphries et al. 2015), ranchers across the USA kill an estimated 500,000 coyotes per year [56] (Flores 2016), whilst the USDA Wildlife Services killed 2.6 million animals (including the ‘unintentional deaths’ of 23 black bears) in 2018 alone, and in Australia, 5,000,000 rabbits died of myxomatosis in the two years after the disease was introduced in the early 1950s. Lethal and non-lethal management methods both exist, but historically and contemporarily killing is the predominant approach. Wildlife management is likely to be more effective when based on an understanding of the behaviour and wider biology of target animals [57] (Baker et al. 2007), but research on such methods has often not progressed beyond experimental, such as with learned food aversions [58,59,60,61,62] (Cox et al. 2004, Macdonald and Baker 2004, Baker et al. 2005, Baker et al. 2008, Baker and Macdonald 2015a).

Commonly used management methods include shooting, poisoning, live and kill trapping, exclusion and deterrents [24] (Littin 2010), which all have clear capacity to impact the welfare of target animals [37,43] (Baker et al. 2022, Baker et al. 2016). Even where killing is effective, non-lethal approaches may sometimes work equally or better, as in the case of protecting livestock from jackals or cheetah [63,64,65] (Marker et al. 2005, 2010, McManus et al. 2015). Non-lethal management may provide advantages not only in terms of effectiveness and sustainability, but also welfare, conservation and ethics [66,67] (Baker and Macdonald 1999, Petracca et al. 2019). However, the accountancy of pros and cons must be meticulous and unbiased. For example, installing rabbit fencing may have a greater welfare disbenefit than well-conducted shooting [37] (Baker et al. 2016), live trapping rats may have a worse welfare impact than killing them with high quality snap traps [43] (Baker et al. 2022), and live traps sold for killing European moles badly compromise mole welfare [68,69] (Baker and Macdonald 2012; Baker et al. 2015). In order properly to incorporate welfare concern into decision making about wildlife management it is necessary to conduct formal assessments of the relative welfare impacts produced by management methods [37,70] (Baker et al. 2016, Allen et al. 2019).

### Welfare in wild Norway Rat Management

Rats are considered significant pests due to their negative impacts on human and animal health, agriculture, property, and the environment [71,72,73,74] (Macdonald et al., 1999; Meerburg et al., 2009; Battersby, 2015; Lambert et al., 2017). However, the use of low-welfare management methods for rats is considered a paradox, as they would not be tolerated for most other species [38,75,76,77,78] (Mason and Littin, 2003; Pesticide Safety Directorate, 1997; Buckle and Smith, 2015; Schlötelburg et al., 2021; Dubois et al., 2017). Such methods include anticoagulant rodenticides, cholecalciferol poisoning, glue traps, snap traps, and cage trapping followed by killing using a concussive blow to the head [38,43,79] (Baker et al., 2022; Mason and Littin, 2003; Frantz and Padula, 1983). A recent study assessed the welfare impacts of six rat management methods and ranked them based on their welfare impact [43] (Baker et al., 2022). The study found that anticoagulant and cholecalciferol poisoning and non-toxic cellulose baiting had the greatest negative welfare impacts, followed by glue trapping, with concussive killing having mild to moderate impact for seconds to minutes. Cage trapping followed by concussive killing had moderate to severe impacts for hours, and the impact of snap trapping was highly variable. The study concluded that globally, rodent control is among the most significant of deliberate human activities affecting animal welfare. The authors recommended improving communication on how to minimise avoidable negative welfare and emphasised that avoiding problems arising in the first place is the best way to protect animal welfare in wildlife management [43] (Baker et al., 2022), including practices such as rodent-proofing to prevent populations from becoming established.

## 3. Impacts of Biodiversity Conservation Practice on Animal Welfare

Biodiversity is made up of genes, species and ecosystems [80] (Norse et al. 1986). Species ‘death rates’ exceed species ‘birth rates’ by about a thousand times [81] (Pimm et al. 2014), making this vast biodiversity loss “*the most severe environmental challenge of the age*” [80,82] (Norse et al. 1986, Pimm 2021). Practitioners of biodiversity conservation differ in their motivations [22] (Vucetich et al. 2021), and the extents to which they include animal welfare in their considerations, but it is the case that animal welfare is impacted not only by the threats to biodiversity, but also by conservationists’ attempts to ameliorate those threats.

As populations consist of individuals, all physical conservation actions are likely to have welfare impacts, for individual target and potentially also non-target animals. How to plan and execute these interventions is a cutting-edge topic for conservation research, e.g., the killing of invasive American mink in the UK [83,84] (Bonesi et al. 2007, Harrington et al. 2009), New Zealand’s drive to trap and eradicate mustelids, rats and possums [85] (Department of Conservation 2021) aiming to render the islands predator-free by 2050, and globally there are efforts to conserve dwindling island populations of endangered bird and reptile species by poisoning rats with non-selective poisons such as the anticoagulant poison brodifacoum [19] (Cowan and Warburton 2011). The list of island invasives extends far beyond feral domestics such as Galapagos goats, dogs and cats to the Hebridean hedgehogs and Caribbean mongooses being controversially weeded out. Even seemingly benign, non-lethal, conservation actions, such as the rehabilitation and release of injured animals, may cause fear, pain and distress to be offset against the possible success of the intervention.

How should the welfare impacts of conservation interventions be treated? Mathews [86] (2010a) offered a consensus statement by specialists who agreed that animal welfare impacts should be mitigated where possible, with the welfare of all individual sentient wild animals given equal concern. How is welfare evaluated in conservation practice? Sometimes at the population level, using variables related to the physical state or biological function of animals, e.g., survival or reproductive success [21] (Beausoleil et al. 2018). Other evaluations consider the physiological or health status of populations or individuals in more detail, e.g., body condition, weight, injury [87,88] (Woodroffe et al. 2005, Gelling et al. 2012), while blood, saliva or faecal samples may be analysed to assess immune function or ‘stress’ [89,90,91] (Moorhouse et al. 2007, Gelling et al. 2009, Narayan 2013). For example, the Leucocyte Coping Capacity (LCC) provides a quantitative comparison of the stressfulness of using, or not using, handling cages and a short-journey to transport captured badgers to a handling facility for examination [92,93] (McLaren et al. 2003; Huber et al. 2019). However, animals’ mental experiences (‘feelings’) are central to understanding animal welfare and insight into those may best be gauged from the intensity and duration of negative experiences, e.g., breathlessness, pain, thirst and hunger. It seems obvious that thorough and transparent welfare assessments should be amongst the various dimensions to be evaluated when making decisions about conservation actions [21] (Beausoleil et al. 2018).

The welfare impacts of some conservation actions have been assessed in this way in the UK, New Zealand and Australia—for example, Fisher and colleagues applied the Sharp and Saunders model [17] (Sharp and Saunders 2011) to assess the relative welfare impacts of a range of toxic agents, used in New Zealand, to kill a number of introduced target and non-target mammals, including possums, rodents, stoats, ferrets and feral cats [94] (Fisher et al. 2008). They concluded that lethal anticoagulant poisoning had the greatest impact on welfare, while 1080 (sodium fluoroacetate) and phosphorus produced intermediate impacts and cyanide (used for possum control) had the least welfare impact.

### Welfare in Conservation Translocations

As with our case study of rat control in the context of agriculture, here, in the context of endangered species conservation, this study explores the case of *conservation translocation*, the intentional human-mediated movement and release of a living organism where the primary objective is a conservation benefit [95] (IUCN/SSC 2013). Translocations may be used to augment existing wild animal populations, create new populations, remove predators from vulnerable prey or remove vulnerable predators from conflict situations. The animals released might be wild caught or captive bred. These actions are effectively ‘forced dispersals’ [96] (Swaisgood 2010) and the potential for animal welfare impacts is clear, from the sourcing of animals to well beyond the time of their release. Even translocation programmes that are considered ‘successful’ often lead to suffering and death among released animals [86] (Mathews 2010a), as well as affecting resident animals of the same and other species [23] (Sainsbury et al. 1995). There is obvious scope for conflict between conservation and welfare interests in translocations [86] (Mathews 2010a).

Where wild animals are live trapped or transported, they are likely to experience stress, hunger, thirst, environmental challenge and behavioural restrictions and their welfare will be compromised by fear, distress, anxiety and frustration [37,43,44,92,96,97] (McLaren et al. 2003, Montes et al. 2004, Swaisgood 2010, Harrington et al. 2013, Baker et al. 2016, Baker et al. 2022). It is a basic tenet that traps should therefore be inspected regularly to minimise the time animals spend in them [43,98] (Proulx et al. 2020, Baker et al. 2022). It is similarly fundamental that live-traps should be as selective as possible for the target species, minimising non-target species capture and consequent welfare impacts [99] (Virgos et al. 2016). It is obvious that wild-caught animals will experience fear and anxiety when brought into captivity [100] (Teixeira et al. 2007) and even captive-bred individuals may suffer in the unnatural confines of captivity. For example, research on physiological measures of stress indicates that it is greater when small mammals are handled [90] (Gelling et al. 2009), and, for water voles being bred for reintroduction, when they are kept in larger groups or smaller cages [89,101] (Moorhouse et al. 2007, Gelling et al. 2010). 

When animals are released into the wild, the welfare of both the released individuals and the resident animals they join may be eroded—a risk that may be reduced by careful choice of the release technique, fitness of animals for release, social groupings and the suitability of the release environment [23,96] (Sainsbury et al. 1995, Swaisgood 2010). Body condition declined in water voles following release and, while stress itself may be difficult to interpret [21] (Beausoleil et al. 2018), indicators of stress including bodyweight: length ratios, plasma corticosteroid levels and neutrophil activation were affected post-release [88,102] (Gelling et al. 2012, Moorhouse et al. 2015b). Few release programmes have tested their release methods and 75–90% of documented reintroductions have failed. Improved social support and using cues to create relationships between the natal habitat or captive environment and the release environment (as attempted for European mink by [103] Maran et al. 2009) might help to improve success rates as well as animal welfare [96] (Swaisgood 2010). Again, formal assessment of welfare impacts could be used to help in decision making about translocation procedures.

**Figure 1:**
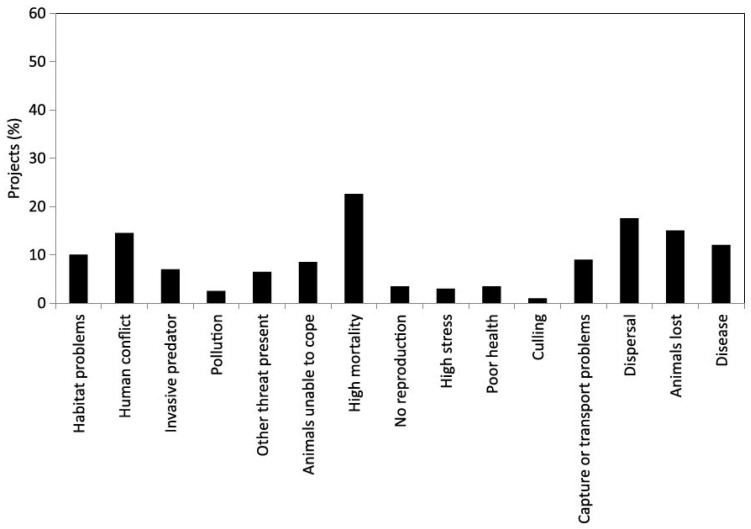
Potential animal welfare problems reported in reintroduction projects. Potential problems are based on the authors’ interpretation of information presented in the literature and do not differentiate between issues affecting one individual animal and those affecting many individuals. Source: Harrington et al. (2013) [44].

Harrington et al. [44] (2013) systematically reviewed the literature on welfare in wildlife reintroductions, analysing 199 projects from scientific articles produced between 1990 and 2008. Problems associated with reintroduction were recorded in 67% of projects, the most common being high mortality, dispersal or loss of animals, disease, and human conflict (Figure 1). A dilemma is that conditions better for welfare prior to release may be less effective preparation for animals for release to the wild, raising the question of how their circumstances in captivity could be modified to improve their prospects [96] (Swaisgood 2010). A potentially longer-term development in wild animal welfare assessment might build on success in using automated high-tech methods to assess the welfare status of whole populations, e.g., a domestic example being the use of Optical Flow to monitor domestic broiler chicken (*Gallus gallus domesticus*) welfare [104] (Dawkins et al. 2017).

**Figure 2:**
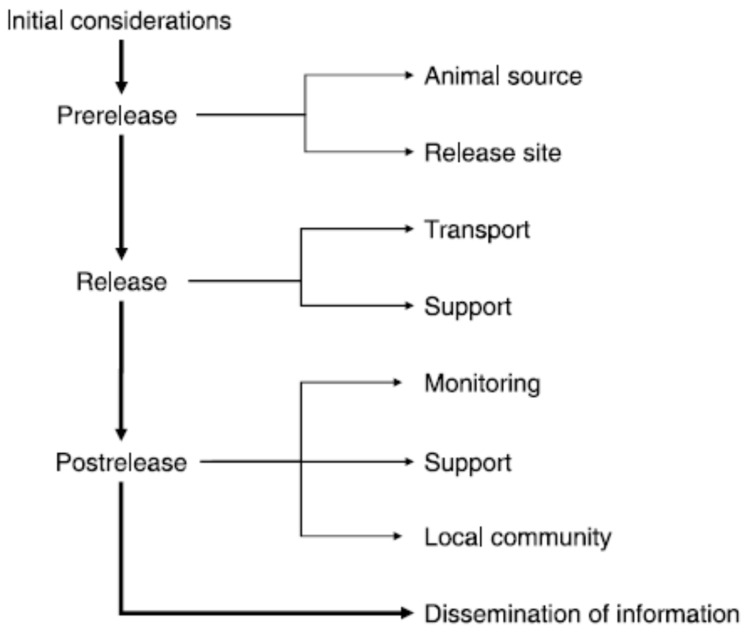
Decision tree outlining animal welfare issues and ethical dilemmas that should be considered in all stages of a reintroduction process. Neither the issues nor the mitigation options are exhaustive; rather, they illustrate the type of decision-making process required to improve animal welfare in reintroduction projects. Given the variable nature of reintroductions among species, habitats, and regions, management solutions will likely be unique to each specific reintroduction. (Note: an expanded version of the decision tree can be found in Appendix A).

As a result of their review, [44] Harrington et al. (2013) developed a decision tree to assist conservation practitioners in identifying welfare considerations at each stage of the reintroduction process (Figure 2). They urge conservationists conducting relocations to report animal welfare issues, describe mitigation actions, and evaluate their efficacy, with the aim of enabling evaluation of common problems and advancing shared strategies for resolving them, e.g., the captive breeding and reintroduction of European mink *(Mustela lutreola)* programme on the Baltic island of Hiiumaa [105] (Maran et al. 2017).

## 4. Other Impacts on Wild Animal Welfare

A diversity of other human activities impacts wild animal welfare, including food production [106] (Hampton et al. 2021), wildlife research [107] (Hawkins 2008), wildlife trade [47,108] (Baker et al. 2013; Zhou et al. 2014), wildlife tourism [4] (Moorhouse et al. 2015a), environmental contamination, urban development and transportation [109] (Fraser 2010). A brief highlight of the intersections between wildlife welfare and wildlife friendly farming, wildlife trade and wildlife tourism follows.

### 4.1. Wildlife Friendly Farming

Effective strategies are urgently needed to balance biodiversity conservation and agricultural production as demands on agricultural lands to produce food and fuel continue to expand. Two strategies proposed for this balance are “land sparing” and “wildlife-friendly farming.” Land sparing manages homogeneous areas of farmland to maximise yields while separate reserves target biodiversity conservation. Wildlife-friendly farming integrates conservation and production within more heterogeneous landscapes. Different scientific traditions underpin the two approaches. Land sparing is associated with an island model of modified landscapes, while wildlife-friendly farming emphasises heterogeneity, resilience, and ecological interactions between farmed and unfarmed areas [110] (Fisher et al. 2008), enhancing animal welfare by providing habitats and reducing disruptions, promoting coexistence within a balanced ecosystem.

Ecological intensification is a means to achieve environmentally sustainable increases in crop yields by enhancing ecosystem functions that regulate and support production. A study conducted in England [111] (Pywell et al. 2015) showed that habitat creation in lower yielding areas at the field edge led to increased yield in the cropped areas of the fields, and this positive effect became more pronounced over six years. As a consequence, yields at the field scale were maintained, and some crops even showed enhanced yields despite the loss of cropland for habitat creation.

### 4.2. Welfare in Wildlife Trade

The international wildlife trade is worth billions of US dollars p.a. [112] (Barber-Meyer 2010), and impacts animal welfare in innumerable ways. However, these impacts have received scant research attention; even CITES appears largely to neglect its welfare control duties [47] (see Baker et al. 2013). The fate of at least 200,000 pangolins p.a. is illustrative of the horrors that occur—extracted from their burrows or tree dens, a surely frightening process often taking hours, pangolins are transported alive before being boiled (some still alive) to remove their scales for use in Chinese traditional medicine [113,114] (D’Cruze et al. 2018, Harrington et al. 2018).

**Figure 3:**
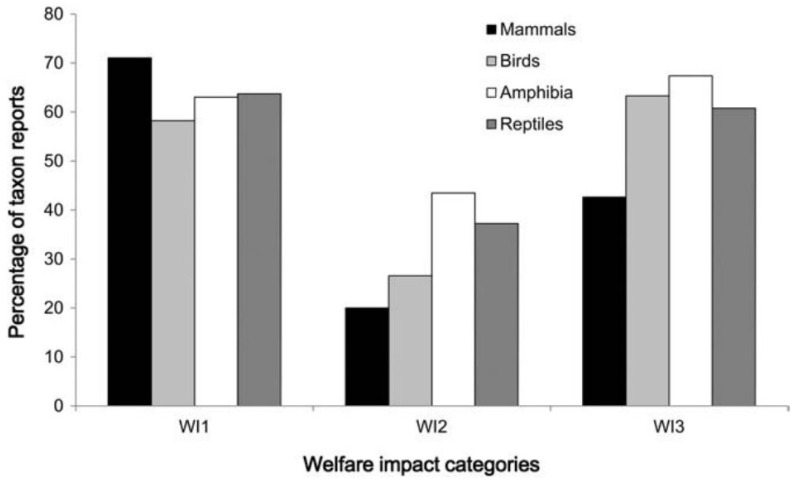
Relationship between taxa (mammals, *n* = 190; birds, *n* = 79; amphibians, *n* = 46; reptiles, *n* = 102) involved in trade and welfare impact categories. Abbreviations: WI1, welfare impact category 1; animal killed on site; WI2, welfare impact category 2: animal captured, transported and killed; WI3, welfare impact category 3: animal used alive. Source: Baker et al. (2013) [47].

In [47] 2013, Baker and colleagues conducted a systematic literature review of welfare in the legal and illegal wildlife trade literature, analysing 292 articles published between 2006 and 2011. The term ‘welfare’ appeared in only 12% of articles, evidence of welfare impacts was rarely recorded and with even fewer suggestions of improvements to ameliorate these impacts. Animals killed on site were most likely to be mammals while those captured alive tended to be birds, amphibians or reptiles (Figure 3). Two salient priorities emerged: (1) greater welfare attention should be given to animals captured and traded alive, and in larger numbers (e.g., birds, reptiles, amphibians), and to those potentially subject to greater impacts through live use (e.g., as pets, see [115] D’Cruze et al. (2015), or entertainment, or farmed for their products, see [116] Bauer et al. (2018) and [117] Williams et al. (2015)); and (2) since evidence suggested that welfare impacts may be under-reported, especially in international, illegal, and wild-caught trade and trade in reptiles, a focus is required in these areas to identify how best to make conservation and welfare improvements there. 

Among traditional medicines, the sourcing of wildlife-origin medicinal materials for Traditional Chinese Medicine (“TCM”) gives rise to an international trade that potentially negatively impacts upon a substantial diversity of animal species: [118] Moorhouse et al. (2021) revealed that the Pharmacopoeia of the People’s Republic of China (PRC) contains 77 medicinal wild animal species, and CITES records from 2008 to 2018 reveal that the PRC exported 23 metric tonnes of medicine derived from these, while the Medical Fauna of China lists 2341 wild animal species, from which more than five metric tonnes of medicines were exported over the same period. Alternatives are sometimes available, e.g., replacing the use in traditional medicine of endangered and sentient vertebrates with sustainably sourced herbal alternatives, an approach seemingly welcomed by a majority of TCM doctors [119] (Moorhouse et al. 2022).

Zoonotic diseases cause millions of human deaths every year. Diseases such as Ebola, severe acute respiratory syndrome (SARS), avian influenza and, most recently and startlingly, COVID-19 cause economic losses at the global level and jeopardise diplomatic relations between countries. Can et al. (2019) [120] showed that mostly the USA and other high-income countries, the largest importers, drive the live animal trade; high-income countries, and not the countries where wildlife diseases and pathogens are more likely to occur, submitted most of the disease reports to the World Organisation for Animal Health. Prevention of illegal trade and re-evaluation of legal wildlife trade are thus also among the priorities needed to tackle these emerging disease. However, although many have stridently recommended banning markets such as the one from which coronavirus disease (COVID-19) probably originally spread, Montgomery and Macdonald [121] (2020) highlight that millions of people around the world depend on markets for subsistence and the diverse use of animals globally defies uniform bans, arguing that the immediate and fair priority is critical scrutiny of wildlife trade.

Demand for exotic pets is a key driver of wildlife trade [47,121] (Baker et al. 2013; Zhou et al. 2015). Animal welfare can be compromised at all stages of the pet trade: capture (e.g., birds being trapped in glue, primate mothers being killed to facilitate capture of young); transport (three animals dying for each one being traded with a consumer); and adjustment to captivity (including radical changes to behaviour, diet, and confinement and exposure to people and other species) [48] (Bush et al. 2014). Moorhouse et al. [122] (2017a) used a novel approach to examine the likely effectiveness of different information on customer demand as means of influencing both the legal and illegal pet trades. Amongst potential disbenefits associated with buying a pet, information on the possible risk of contracting disease significantly affected consumer choices, as to a lesser extent did the risk of legal penalties, reducing respondents’ stated likelihood of buying the proposed pet. In contrast, information on the negative animal welfare and conservation impacts of buying the proposed pet had no significant effect on respondents’ consumer choices. This dismal insight into consumer priorities suggests that information campaigns playing on selfish motivations—the risks of contracting disease (see [123] Macdonald and Laurenson 2006) or punishment—are more likely to be influential than highlighting cruelty or extinction.

### 4.3. Welfare in Wildlife Tourism

Wildlife tourism impacts animal welfare: collectively, the scale of these impacts is enormous. Moorhouse et al. [4] (2015a) identified 24 types of wildlife tourist attractions (WTAs), excluding zoos and consumptive WTAs, which they estimated to be visited by 3.6–6 million tourists globally p.a. They scored the conservation and welfare impacts of each, using scores between −3 and +3. Arriving at a taxonomy of WTA types, six types scored positive or neutral impacts for both conservation and welfare (e.g., various kinds of sanctuaries), while four types scored positively for conservation but negatively for welfare (e.g., gorilla trekking), and 14 scored negatively for both (e.g., civet coffee farms). An estimated 2–4 million tourists p.a. participated in activities likely to have negative welfare and/or conservation impacts.

Moorhouse et al. [4] (2015a) also examined customer reviews of WTAs on the TripAdvisor website (www.tripadvisor.co.uk, accessed on 1 May 2014), finding that tourist ratings correlated only loosely with their assessment of welfare conditions, and on only 6–17% of the reviews did tourists express dissatisfaction with welfare conditions. The authors concluded that tourists tend to be poorly equipped to judge what constitutes acceptable use of wildlife—some may assume that the very existence of a facility is evidence that an informed authority has judged it to be of a suitable standard. Two solutions are apparent, first, well-founded regulation, and second education to inform consumer behaviour with respect to welfare and conservation impacts of WTAs [124] (Moorhouse et al. 2017c). Using online surveys, Moorhouse and colleagues [125] (2017b) found that respondents preferred beneficial WTAs and rejected detrimental WTAs when primed to consider welfare and conservation impacts. They presented their findings to TripAdvisor, who have since stopped selling tickets to attractions involving interactions with captive wild animals or endangered species and created an education portal for wildlife tourist attractions featured on their site (https://www.tripadvisor.com/blog/animal-welfare-education-portal/, accessed on 1 August 2023).

### 4.4. Urbanisation Impacts on the Welfare of Wild Animals

Partecke et al. [126] (2006) found that European blackbirds hatched in a city have a lower stress response than their forest-living counterparts, likely due to genetic differences. This supports the idea that urbanisation changes the stress physiology of animals and creates a shift in coping styles. This reduced stress response is likely necessary for animals that thrive in ecosystems exposed to frequent anthropogenic disturbances, such as urban areas, with implications for their welfare in response to potentially deleterious factors.

## 5. Synergies between Animal Welfare and Biodiversity Conservation

Conservation biology is concerned with wild animals, with success often evaluated at the level of populations, ecological systems and genetic types, within a framework of threats to biodiversity and ecological integrity. Animal welfare science operates at the level of individuals and their groups, and deals with threats to animal health and quality of life [109] (Fraser 2010). Populations consist of individuals, and the processes that underlie population dynamics are emergent properties of the behaviour and fates of individuals. Therefore, the well-being of a population is likely, at least often, to be affected by the well-being of the individuals that comprise it. Despite this likely synergy, the conservation and welfare of wild animals have often been presented as opposing interests [127] (Dubois and Fraser 2013), differing in focus, goals and strategies [44] (Harrington et al. 2013). Beausoleil et al. [21] (2018) depict the relationship between these two facets of concern for wild animals by the characterisation that when conservationists consider animal welfare they tend to focus on an animal’s ‘fitness’, whereas welfare scientists tend to emphasise animals’ ‘feelings’. They conclude that survival or biological function are insufficient measures of welfare in the context of conservation. Considering their shared focus on animals and their shared multidisciplinarity, there is inevitably substantial overlap in the interests of animal welfare scientists and conservation scientists, and opportunities for cooperation; Swaisgood [96] (2010) exemplifies this with the case of captive breeding and reintroduction, where if a programme fails to ensure the health and welfare of animals in captivity prior to release, then the project’s conservation objectives are unlikely to be met. However, there are obvious areas of conflict: an example is the growing concern over animal welfare in island conservation programmes, many of which rely on the eradication of introduced rodents, commonly using very low welfare methods, to safeguard another species [19,43,128] (Cowan and Warburton 2011, Thomas et al. 2011, Baker et al. 2022). In the same vein, even successful reintroduction programmes often result in welfare impacts, including deaths, among released animals [44] (Harrington et al. 2013)—Bull et al.’s [129] (2019) exploration of the restoration of wolves, and with them natural processes such as predation and predator-prey cycles, to Scotland, might be evaluated, inter alia, from the perspective of red deer welfare (balancing disembowelling fangs against biting hunger or the stalker’s bullet).

Fraser [109] (2010) foresees welfare and conservation problems merging as human populations increase; conservation issues are generally associated with welfare concerns, but the converse is less generally true [130] (Blumstein 2010). However, welfare issues are potentially important early indicators of incipient conservation problems. Additionally, social license to operate is an important factor in public acceptance of human interactions with wild animals [131] (Hampton and Teh-White 2019) and since many citizens, at least in Western society, consider *both* welfare and conservation important, interventions to foster both conservation and welfare programmes need to engage stakeholders convincingly.

Beausoleil and colleagues [21] (2018) conclude that scientists from both specialisms should recognise a conceptual framework that integrates both fitness and feelings into a science that they propose be named Conservation Welfare. I agree the necessity, potency and ethical imperative of the integration, although as a conservation biologist and practitioner I have always included animal welfare as an important arrow in the quiver of conservation considerations [132,133,134,135,136] (Macdonald & Boitani 1979, Macdonald and Dawkins 1981, Macdonald 2001, Macdonald et al. 2006a, Macdonald and Willis 2013). In a way compatible with non-anthropocentrism [22,137] (Vucetich et al. 2018a. 2021), the experiences of animals themselves are central to Beausoleil et al.’s [21] (2018) conception of welfare. Batavia et al. [138] (2021) argue that compassion can be characterised ‘as an emotional experience of interdependence and shared vulnerability’ between people and wild animals’. This, they say, highlights conservationists’ responsibilities to individual beings in turn linking into individuals being the foundation of populations, species, and ecosystems, concluding that compassion, thus defined, should form a central virtue of conservation.

Conservation of biodiversity underpins many of the UN’s sustainable development goals (SDGs)—in 2019 the organisation’s Global Sustainable Development Report acknowledged for the first time that improvement of animal welfare was missing from the enumeration of the SDGs, stating: ‘*The clear links between human health and well-being and animal welfare are increasingly being recognized in ethics- and rights-based frameworks. Strong governance should safeguard the well-being of wildlife and domesticated animals with rules on animal welfare embedded in transnational trade*’.

## 6. Balancing Welfare with Other Priorities

Despite the arguments, and everyday intuitions, for accounting for welfare in conservation, there has been little scientific dialogue until recently [137] (e.g., Vucetich et al. 2018a) on the ethical dimensions of conservation [139] (Vucetich and Nelson 2007). Most conservation and welfare scientists would probably agree that (at least) vertebrates can feel pain or fear, and should be considered sentient [140] (Mason 2011). Vucetich et al. (2018a) develop the non-anthropocentric principle to guide conservationists to acknowledge this and the associated intrinsic value of vertebrates (see also [141] Pooley and Redpath 2018 and [142] Vucetich et al. 2018b). An extreme position, that all suffering should be avoided regardless of the conservation cost (i.e., welfare is sacrosanct as a moral imperative), can be aligned with the philosophy of Immanuel Kant and traced back to the teachings of Hinduism and Jainism regarding neither harming nor killing animals. Towards the other end of a continuum of positions, a consequentialist view, while striving to minimise suffering might offset any residual welfare disbenefit against potential conservation benefits. In charting a route to an ethically sound framework for conservation, that incorporates animal welfare and is non-anthropocentric, Vucetich et al. [22] (2021) review these two ethical frameworks, Kantian deontology and the consequentialism or utilitarianism generally associated with Jeremy Bentham and John Stuart Mill. They also introduce a third, yet more venerable, framework, virtue ethics, which emphasises virtues and the Aristotelian quest for well-being termed eudemonia, ‘a word that while not readily translated into English, refers to a kind of human flourishing, a rich sense of happiness that transcends hedonism’.

A minimal consensus might be that non-human animals should be harmed no more than necessary, but, to what extent can or should the welfare of an individual be sacrificed for a wider benefit—or vice versa [132,133] (Macdonald and Boitani 1979, Macdonald and Dawkins 1981)? In 2001, Macdonald and Tattersall [143] sought to develop an ideology for 21st-century conservation advocating an ethic they termed respectful engagement. Nowadays, in many developed countries, the reality of living with wildlife is increasingly distant and both the thrill and the anguish of that reality may be muted. Nonetheless, amongst those that do experience nature directly there are some that disavow the extreme of brutal utilitarianism but nonetheless do not consider every life sacred. Their ethics can embrace a fervent desire to conserve and foster with an acceptance of a need sometimes to manage and to use, but for each of those engagements to be respectful (the anguish of killing wonderful, but abundant, predators to conserve wonderful, but imperilled, prey is vividly expressed by Colwell [144] (2021)). For those for whom the notion of respect is too woolly or mystical, Macdonald and Tattersall [143] (2001) suggest it might be characterised as “*taking the responsibility to be aware of all aspects of the issue*”. The holistic approach of transdisciplinary conservation [2] (Macdonald 2019) strives to capture “*all aspects of the issue*”, which inform the question of balance to be executed through ‘*respectful engagement*’ [143] (Macdonald and Tattersall 2001).

Much of modern conservation concerns conflict between wildlife and people, and much modern thinking about human–wildlife conflict [145] (e.g., Pooley et al. 2017) is congruent with the sentiments favouring transdisciplinarity and respectful engagement that light the road ahead. Vucetich et al.’s [137] (2018a) exploration of the intersection of conservation and social justice around human–wildlife conflict proposes: ‘*Humans should not infringe on the well-being of others (including other humans, large carnivores, or other parts of nature with intrinsic value) any more than is necessary for a healthy, meaningful life.*’. Recognising that the injunction to avoid inflicting negative animal welfare may sometimes be infeasible, their second proposal is that: ‘*When the ability to live a healthy, meaningful life genuinely seems to infringe on the wellbeing of some intrinsically valuable element of nature (such as large carnivores), then the just solution will less often be found in depriving large carnivores and more often be found in rectifying an unjust inequality among humans’.*

When planning any wildlife intervention, there is a need to balance the likely animal welfare costs not only against the benefits of the primary goal of the intervention (wildlife conservation, management or other purposes), but also against a diversity of other important factors, e.g., financial cost, effectiveness, cost-effectiveness, non-target welfare, safety for user and other people, ease of application, public acceptability, and environmental impacts [41,146] (Littin et al. 2014, Baker et al. 2020). This calculation should be situation-specific and undertaken anew for each intervention [146,147] (Schwartz et al. 2018, CRRU UK 2021). Breaking into the cycle of prevention versus cure, if an eradication has been necessary, it should be followed by the necessary biosecurity to prevent recurrence [148] (Pierce and Teroroko 2011).

Two frameworks exist to guide actions in wildlife management. Dubois and colleagues [78] (2017) proposed a set of inter-dependent and step-wise international consensus principles for ethical wildlife management: (1) modify human practices that cause the problem where possible and develop a culture of coexistence; (2) justify the need for control, with evidence that significant harms are being caused to people, property, livelihoods, ecosystems, and/or other animals; (3) have measurable outcome-based objectives that are clear, achievable, monitored, and adaptive; (4) predictably minimise animal welfare impacts on the fewest animals possible; (5) consider community values and scientific, technical and practical information; (6) include long-term systematic management; and (7) base control on the specifics of the situation rather than negative labels (pest, overabundant, invasive) applied to the target species. This iterative, adaptive management approach closely parallels the so-called BICS (Biodiversity Impacts Compensation Scheme) proposed by Macdonald [149] (2000), and elaborated by Macdonald and Sillero-Zubiri [150] (2004), to progressively whittle away the extent of human–wildlife conflict, minimising the intractable residue requiring lethal or non-lethal intervention or compensation. The second framework is a ‘risk hierarchy’ devised by the Campaign for Responsible Rodenticide Use (CRRU), which is intended to be central to decision making regarding a rodent management strategy for any site. Additionally, if it works for rodents, why not lions and elephants? The hierarchy presents stepwise measures for preventing or removing a rat or mouse population. It emphasises proofing methods and removal of harbourage, and when lethal management is necessary, using the least severe method considered to be effective, as well as minimising non-target and environmental risks [147] (CRRU UK 2021).

In conservation, a number of decision support frameworks exist to aid practitioners in planning conservation interventions while increasing planning rigor, project accountability, stakeholder participation, transparency in decision making and learning. Schwartz and colleagues [146] (2018) recommend adapting frameworks to suit specific needs, for example, choosing and adapting tools from different frameworks to maximise utility; any practitioner who, similar to me, considers welfare a key element of conservation decision making, could readily incorporate animal welfare tools. For example, one framework, structured decision making, can be used to identify which actions might most efficiently achieve competing objectives [151] (Gregory et al. 2012), and animal welfare assessment models [17] (Sharp and Saunders 2011) or systematic reviews (e.g., [47] Baker et al. 2013) could be added. Conservation scientists and practitioners may differ in the weighting they implicitly give animal welfare, but it is clear that in all the foregoing frameworks animal welfare could be explicitly considered, along with other priorities, in planning every conservation intervention.

## 7. Conclusions

Wild animal welfare is finally beginning to attract the research attention it warrants, but progress to develop tools to quantify welfare impacts, means of mitigating them and, most of all, clarity on ethical dilemmas, that might influence both policy and practice, have all been slow. I consider that there is a moral obligation for humans to minimise their impacts on non-human animal welfare, regardless of that animal’s perceived status, whether it be considered ‘pest’, ‘feral’, ‘introduced’, ‘abundant’ or ‘rare’.

There is a great deal of overlap in the interests of animal welfare and conservation scientists, and much overlap too between the practitioners and advocates for, and supporters of, these two emphases on animal well-being. Therefore, there is much to be gained by both through collaboration and knowledge-sharing and by incorporating consideration of welfare and conservation into policy, guidelines, legislation and practice. Attention should focus on animals’ ‘feelings’ as well as their ‘fitness’. Impacts associated with non-lethal interventions merit no less attention than those associated with killing non-human animals, and impacts that happen repeatedly, or are prolonged, need greater consideration.

Above all, mindful of the adage that prevention is better than cure, humans should strive to change their behaviour to avoid situations where wild animal welfare is jeopardised, and where that cannot be avoided their engagement with individual non-human animals should be respectful.

## Data Availability

Data sharing not applicable.
